# Description of four new *Gunungiella* species from central Vietnam (Trichoptera, Philopotamidae)

**DOI:** 10.3897/zookeys.1287.191678

**Published:** 2026-08-04

**Authors:** Kjell Arne Johanson, Hong Thai Pham

**Affiliations:** 1 Zoology Department, Swedish Museum of Natural History, Box 50007, 10405 Stockholm, Sweden Mientrung Institute for Scientific Research, Vietnam National Museum of Nature, Vietnam Academy of Science and Technology Hanoi Vietnam; 2 Mientrung Institute for Scientific Research, Vietnam National Museum of Nature, Vietnam Academy of Science and Technology, 18 Hoang Quoc Viet Street, Hanoi, Vietnam Zoology Department, Swedish Museum of Natural History Stockholm Sweden

**Keywords:** Caddisflies, central Vietnam, identification key, new record, new taxa, running water, taxonomy

## Abstract

Four previously unknown species of *Gunungiella* Ulmer, 1913 are herein described from the Bach Ma National Park in the Phu Loc commune of Hue City, located in central Vietnam: *G. phulocensis***sp. nov**., *G. bacmaensis***sp. nov**., *G. vietnamensis***sp. nov**., and *G. hueensis***sp. nov**. These species are considered rare and are only encountered as singletons. The specimens were collected in March and April 2023 using Malaise traps and light traps, which were strategically positioned along stream banks. The habitats are permanent, medium-sized, clear-water streams with slow to fast-flowing currents and stony bottoms, surrounded by dense forests on steep slopes. A key for the identification of male individuals of the thirteen known *Gunungiella* species in Vietnam is provided.

## Introduction

Together with Stenopsychidae Martynov, 1924, Philopotamidae Stephens, 1829 are classified in the superfamily Hydropsychoidea (Malm et al. 2013). The two families were hypothesized to diverge about 138 million years ago (Malm et al. 2013). By 2022, the Philopotamidae included more than 1600 species classified in 22 recent genera (Morse 2026). With more than 950 species, the genus *Chimarra* Stephens, 1829 in the subfamily Chimarrinae Rambur, 1842, is the largest in terms of described species. Most genera in the family are restricted to only one biogeographical region, and the present knowledge (Morse 2026) indicates that the Oriental region holds approximately 630 species in eight genera, of which four genera (*Dolomyia* Schmid, 1991, *Dolopsyche* Schmid, 1991, *Edidiehlia* Malicky, 1993 and *Gunungiella* Ulmer, 1913) are endemic to the region; the first three genera are monotypic. Among these genera is *Gunungiella* Ulmer, 1913, which, as of this report, comprises 86 described species. According to Johanson et al. (2023) nine species were previously recorded from Vietnam, all restricted to only one area, indicating high level species endemism: *Gunungiella arinada* Malicky, 1995 is only known from the Vinh Phuc Province; *G. bongai* Oláh & Malicky, 2010, *G. cao* Oláh & Malicky, 2010, *G. dung* Oláh & Malicky, 2010 and *G. lencao* Oláh & Malicky, 2010 from the Lam Dung Province; *G. obendio* Oláh & Malicky, 2010 from the Ninh Bing Province; *G. sosau* Oláh & Malicky, 2010 from the Bac Kan Province; *G. guni* Malicky, 2009 from the Tuyen Quang Province, and *G. thuba* Oláh & Malicky, 2010 from the Hoa Binh Province. As far as we know, all species in Vietnam are associated with spring brooks, streams and waterfalls (Johanson et al. 2023).

In this paper, four new species are described, together with documentation of a fifth species, representing the first records of the genus *Gunungiella* from the Bach Ma National Park and Hue City.

## Material and methods

The material consists of male specimens collected using light and Malaise traps placed at streams in the Bach Ma National Park in Hue City in March-April 2023. The habitats consisted of clear-water streams within dense forests surrounded by steep mountains. The specimens were preserved directly in 80% ethanol along with other insects collected during the same event. Specimens of Trichoptera were initially sorted and identified to the family and genus levels at the Swedish Museum of Natural History (**SMNH**), with identification primarily based on the work of Malicky (2010). Before detailed examination and illustration, each abdomen was macerated in Proteinase K and extracted for DNA simultaneously. DNA extraction was performed using the QIAamp® DNA MicroKit (QIAGEN). The abdomens were dehydrated in absolute alcohol and temporarily suspended in Euparal on a microscope slide. Illustrations of genitalia were prepared using a Leitz Ortholux II light microscope equipped with a drawing tube. These illustrations were then scanned and finalized using Adobe® Illustrator® ver. 28.7.1 and Photoshop® ver. 25.11.0. After examination and illustration, the abdomens were returned to 80% ethanol with the rest of the specimen. Forewing photographs were captured using a Nikon DS Ri2 digital camera mounted on a Nikon SMZ25 stereo microscope. Wing and genital measurements were obtained using NIS Elements D ver. 5.10.01 software. All drawings and photographs were edited and finalized in Adobe Photoshop. For descriptions based on a single specimen, the species were confirmed to be distinct from all other Vietnamese species, as well as some other Oriental species, based on a combination of morphological features and DNA sequences. In the descriptions, the genital terminology follows Nielsen (1957), and wing venation terminology follows Mosely (1939).

The holotypes are deposited in the Vietnam National Museum of Nature (**VNMN**), Vietnam Academy of Science and Technology (**VAST**), Hanoi, Vietnam. The male non-type of *Gunungiella sosau* Oláh & Malicky, 2010 is deposited in the Swedish Museum of Natural History (**NHRS**).

## Taxonomy

### Gunungiella

Taxon classificationAnimaliaTrichopteraPhilopotamidae

Ulmer, 1913

210F6188-A8AB-5150-91C1-CD1C7ADE39E7

Gunungiella Ulmer, 1913: 82.

### Gunungiella vietnamensis sp. nov.

Taxon classificationAnimaliaTrichopteraPhilopotamidae

DCF90248-35B6-57C7-82E9-58BB96CC20D1

https://zoobank.org/13C4A373-AF5C-44C3-801D-87870A4B351F

Figs 1, 5–9

#### Type material.

***Holotype***. Vietnam • ♂; Thua Thien Hue Province, Phu Loc District, Bach Ma National Park, small river, 16.2292°N, 107.8481°E, 362 m asl, 22W light trap, 22.iii.2023, loc#VN23-02, leg. KA Johanson & T Vu, VNMN in alcohol.

**Figures 1–4.**
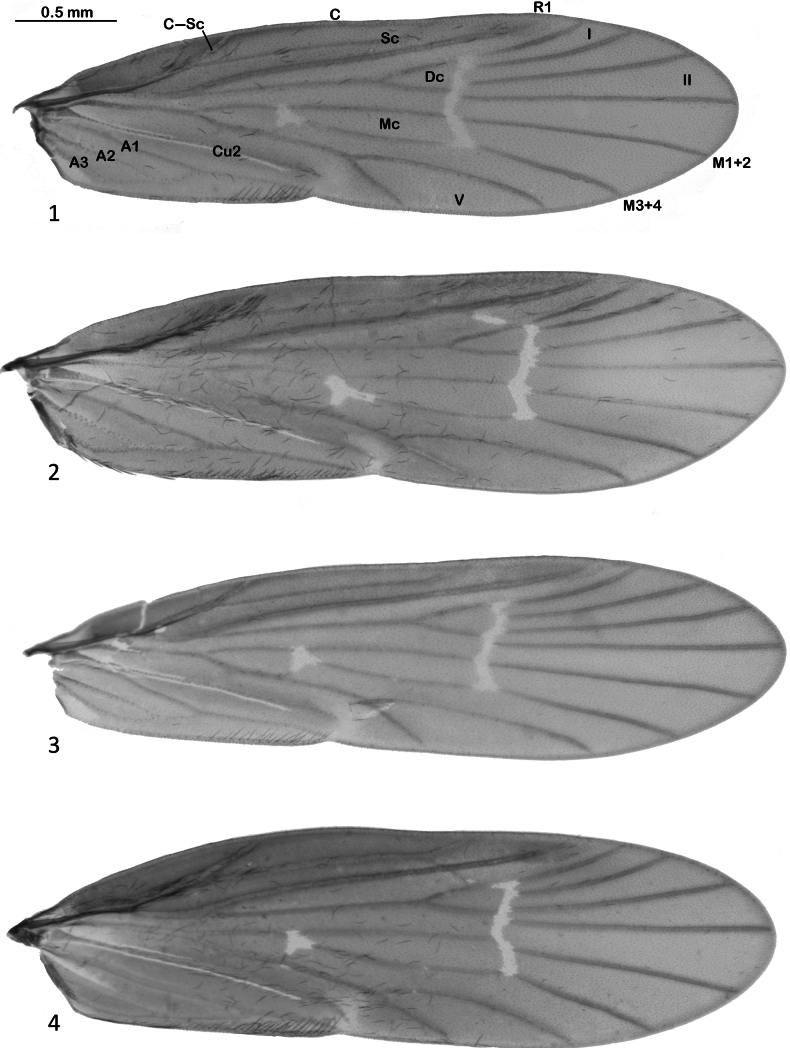
Right forewing of the holotype of four new *Gunungiella* species. **1**. *G. vietnamensis* sp. nov.; **2**. *Gunungiella bacmaensis* sp. nov.; **3**. *Gunungiella hueensis* sp. nov.; **4**. *Gunungiella phulocensis* sp. nov. Scale bar: 0.5 mm; applies to all wings. The terminology on the veins is given in Fig. 1.

#### Diagnosis.

The male genitalia of *Gunungiella vietnamensis*, new species, exhibit a notable similarity to those of *Gunungiella hueensis*, new species, from Bach Ma National Park in Vietnam, and to *G. bongai*, particularly in the bifurcate harpagones as seen in lateral view, and segment IX with longitudinal lateral spiny branches laterally to base of tergum X. *Gunungiella vietnamensis*, new species, is separated from *G. bongai* in lateral view of the genitalia by the sharply pointed apex of both the dorsal and ventral branches of the harpagones; the apex of the dorsal branch of the harpagones orienting posterodorsally, not posteroventrally; the coxopodites are clearly higher than the harpagones, not of the same height as in *G. bongai*; segment IX has one pair of longitudinal, spine-like processes near base of tergum X, not two pairs. *Gunungiella vietnamensis*, new species, is separated from *G. hueensis*, new species, in lateral view of the genitalia by the apex of each branch of the harpagones being sharply pointed instead of rounded; the harpagones are about half as high as the coxopodites, not almost as high as the coxopodites; the pair of longitudinal, spine-like processes on segment IX originate clearly anteriorly of base of tergum X and curving mesally along their length, not originating near base of tergum X and not straight; and the almost ventrally curving phallus has four internal spines, while the phallus of *G. hueensis* is almost straight and has two internal spines.

#### Description.

**Figures 5–9.**
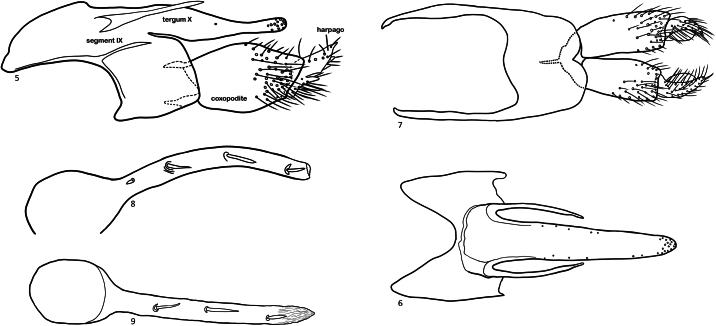
Male genitalia of the holotype of *Gunungiella vietnamensis* sp. nov.; **5**. Lateral view; **6**. Dorsal view; **7**. Ventral view; **8**. Phallus, lateral view; **9**. Phallus, ventral view.

Forewing (Fig. 1) 3.4 mm (*N* = 1), membrane pale brownish, veins dark brown. Fork 1, 2 and 5 present; fork 1 originating distally at anterior corner of Dc; fork 2 originating slightly more basally than fork 1; crossvein R1—R2+3 not hyaline, ending distantly on Dc; Dc about half as long as Mc and 0.12× length of forewing. Base of fork 1, fork 2 and crossvein R3—R4 hyaline. Crossvein R—M hyaline, crossvein M1+2—M3+4 forming hyaline continuation of crossvein R—M; base of Mc forming almost triangular hyaline area. Cu1a almost straight except proximally and distally; distal part of Cu2 and arc hyaline. Posterior margin with row of anteriorly oriented setae in row about as long as Dc. Genitalia (Figs 5–9): In lateral view, segment IX divided into high anterodorsal part and high posteroventral part not separated from each other but forming continuous pleurite; anterodorsal part almost V-shaped; posteroventral part of segment IX slightly shorter than anterodorsal part, as long and high as coxopodites in lateral view (Fig. 5). In lateral view, coxopodites originating as linear continuation of posteroventral plate of segment IX, with posterodorsal corner produced posteriorly; dorsal and ventral margins convex; posterior margin almost straight (Fig. 5); in ventral view, with each side diverging posteriorly, inner margin narrowly V-shaped (Fig. 7). Harpagones about one-third as long and about half as high as coxopodites, divided into high, long dorsal branch with pointed apex, and short ventral branch with pointed apex; dorsal branch oriented posterodorsally, ventral branch oriented posteriorly; in ventral view dorsal part rounded, ventral part arched (Fig. 5). Tergum X oriented posteriorly, narrowing distantly towards mid-length in lateral view, apex rounded (Fig. 5); in dorsal view (Fig. 6) uniformly and slightly narrowing along its length; thick setae absent. Phallus (Figs 8, 9) as long as sum of segments IX and tergum X, curving ventrally along its length; with four sclerites located at narrow part; anterior third globular, posterior two-thirds almost uniformly slender, parallel-sided, one-quarter as high as anterior part (Fig. 8).

#### Etymology.

*Gunungiella vietnamensis* was named after Vietnam, the type country of the new species.

### Gunungiella bacmaensis sp. nov.

Taxon classificationAnimaliaTrichopteraPhilopotamidae

4F906EE5-837F-506C-A878-327015FCB923

https://zoobank.org/F72C4CDA-8E86-42A2-A320-47E2E16F33F7

Figs 2, 10–14

#### Type material.

***Holotype***. Vietnam • ♂; Thua Thien Hue Province, Phu Loc District, Bach Ma National Park, behind water pump house, small stream with bottom of large and small stones, 16.1956°N, 107.8579°E, 1306 m asl, 22W light trap, 24.iii.2023, loc#VN23-11, leg. KA Johanson & T Vu, Vietnam, Thua Thien Hue, VNMN in alcohol.

#### Diagnosis.

The male genitalia of *Gunungiella bacmaensis* sp. nov. exhibit exclusive characters not expressed in other species in the genus. A clearly distinguishing character is the coxopodites, each with an upper and a ventral face separated by a lateral inwardly oriented longitudinal folding from mid-length of each coxopodite; each upper surface with posteriorly pointing posterodorsal corner, each lower surface with the harpago situated at the posterior end. The dorsal part of segment IX resembles that of *G. bongai* in that two pairs of posteriorly oriented branches originate from the posterior part and run in parallel with tergum X. *Gunungiella bacmaensis* new species is distinguished from *G. bongai* in that the paired ventral branch of segment IX is ventrally curving distally and exceeds the apex of tergum X, while in *G. bongai* the ventral branch is straight and does not reach the apex of tergum X.

#### Description.

**Figures 10–14.**
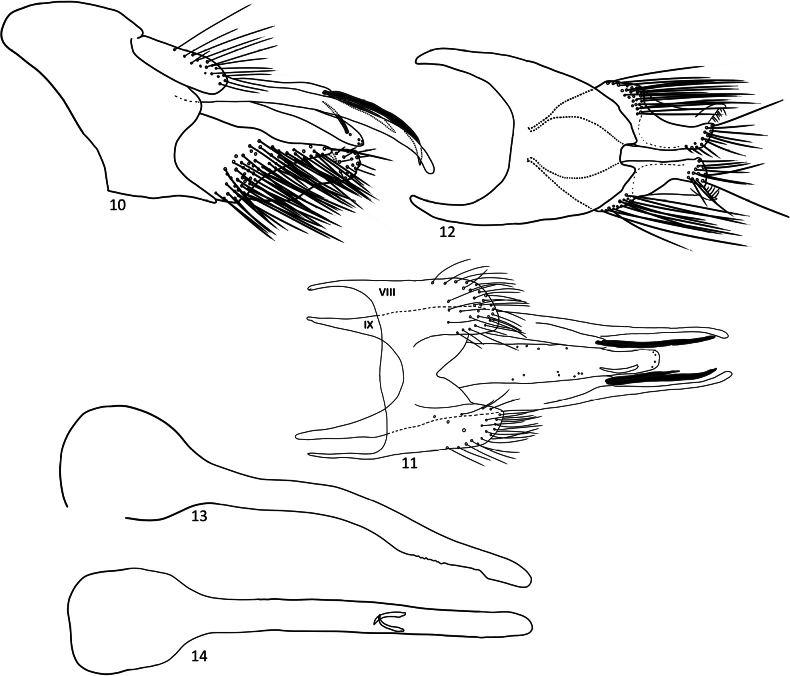
Male genitalia of the holotype of *Gunungiella bacmaensis* sp. nov.; **10**. Lateral view; **11**. Dorsal view; **12**. Ventral view; **13**. Phallus, lateral view; **14**. Phallus, ventral view.

Forewing (Fig. 2) 3.7 mm (*N* = 1), membrane pale brownish, veins dark brown. Fork 1, 2 and 5 present; fork 1 originating distally at anterior corner of Dc; fork 2 originating more basally than fork 1; crossvein R1—R2+3 hyaline, ending at distal half of Dc; Dc about half as long as Mc and 0.11× length of forewing. Base of fork 1, fork 2 and crossvein R3—R4 hyaline. Crossvein R—M hyaline, crossvein M1+2—M3+4 forming hyaline continuation of crossvein R—M; base of Mc forming almost Y-shaped hyaline area. Cu1a almost straight except proximally and distally; distal part of Cu2 and arc hyaline. Posterior margin with row of anteriorly oriented setae in row about 2× longer than Dc. Segment VIII partly hiding segment IX in lateral and dorsal view; with pair of long setose lobes in lateral view; in dorsal view, lobes clearly separated, each almost parallel-sided. Genitalia (Figs 10–14): In lateral view, segment IX with anterodorsal and posteroventral parts not divided, forming continuous plate with only slightly concave anteroventral margin; posterior margin deeply concave before coxopodites (Fig. 10). In ventral view, segment IX with wide U-shaped anterior incision stretching to mid-length of segment IX (Fig. 12); posterior half of segment IX softly narrowing posteriorly into minute U-shaped incision at posterior margin between base of coxopodites (Fig. 12). Pair of bifurcated processes originating below lobes of segment VIII (Figs 10, 11); dorsal branch parallel with ventral branch, each with pair of dark megasetae originating from apex and the longest almost reaching apex of lower branches; lower branches slightly curving ventrally along their length (Fig. 10). Coxopodites, lateral view, each with upper and ventral face separated by a lateral inwardly oriented longitudinal folding from mid-length of each coxopodite; upper surface with posteriorly pointing posterodorsal corner, lower surface with harpago situated at apex. Harpagones less than one-fifth the length of the coxopodites, almost invisible behind dense setae in lateral view (Fig. 10); in ventral view forming small posterolateral lobes above apex of coxopodite (Fig. 12). Tergum X straight, slightly oriented posteroventrally, slender along its length in lateral and dorsal view (Figs 10, 11), in lateral view apex as thick as rest of segment; in dorsal view, posterior one-fourth slightly narrower than basal three-fourth, apex reaching apex of harpagones (Fig. 10); single stout seta present on left side near apex. Phallus (Figs 13, 14) slightly longer than sum of segments IX and tergum X; anterior two-fifths almost globular in lateral view, rectangular in ventral view; posterior three-fifths slender, parallel-sided, with pair of small, slightly curving sclerites located shortly after mid-length of phallus (Fig. 14).

#### Etymology.

*Gunungiella bacmaensis* was named after Bach Ma National Park, from where the holotype was collected.

### Gunungiella hueensis sp. nov.

Taxon classificationAnimaliaTrichopteraPhilopotamidae

8E86CC0E-9386-5C46-B359-A33E47153260

https://zoobank.org/BDA234C0-C349-49E2-97A1-3520D9C82016

Figs 3, 15–19

#### Type material.

***Holotype***. Vietnam • ♂; Thua Thien Hue Province, Phu Loc District, Bach Ma National Park, behind water pump house, small stream with bottom of large and small stones, 16.1956°N, 107.8579°E, 1306 m asl, 22W light trap, 24.iii.2023, loc#VN23-11, leg. KA Johanson & T Vu. Vietnam, Thua Thien Hue, VNMN in alcohol.

#### Diagnosis.

The male genitalia of *Gunungiella hueensis*, new species, exhibit a notable similarity to those of *Gunungiella vietnamensis*, new species, from Bach Ma National Park in Vietnam, and to *G. bongai* Oláh & Malicky, 2010 from the Lam Dong Province in Vietnam, particularly in the bifurcate harpagones as seen in lateral view, and segment IX with longitudinal lateral spiny branches laterally to base of tergum X. *Gunungiella hueensis*, new species, is separated from *G. bongai* in lateral view of the genitalia by the apex of the dorsal branch of the harpagones being more than three times higher than the ventral branch, not twice as high; apex of dorsal branch of the harpagones pointing posterodorsally, not posteroventrally; the coxopodites are clearly higher than the harpagones, not of the same height; segment IX has one pair of longitudinal, spine-like processes near base of tergum X, not two pairs; and the phallus is almost straight in lateral view, not smoothly curving. *Gunungiella hueensis*, new species, is separated from *G. vietnamensis*, new species, in lateral view of the genitalia by the apex of each branch of the harpagones being rounded instead of sharply pointed; the harpagones are almost as high as the coxopodites, not half its height; the pair of longitudinal, spine-like processes on segment IX are straight and originate near base of tergum X, not curving mesally and originating anteriorly of base of tergum X; and the almost straight phallus has two internal spines, while the phallus of *G. vietnamensis* is smoothly curving ventrally, and has four internal spines.

#### Description.

**Figures 15–19.**
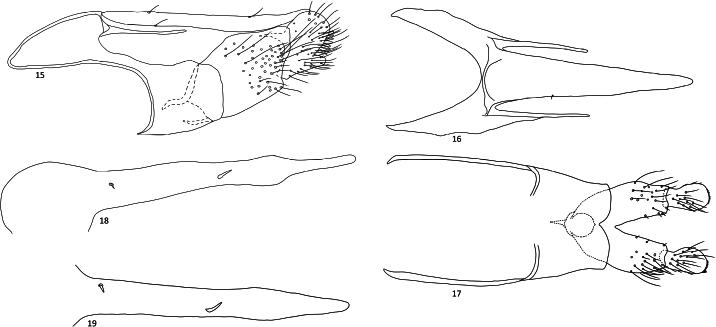
Male genitalia of the holotype of *Gunungiella hueensis* sp. nov.; **15**. Lateral view; **16**. Dorsal view; **17**. Ventral view; **18**. Phallus, lateral view; **19**. Phallus, ventral view.

Forewing (Fig. 3) 3.6 mm (*N* = 1), membrane pale brownish, veins dark brown. Fork 1, 2 and 5 present; fork 1 originating distally at anterior corner of Dc; fork 2 originating more basally than fork 1; crossvein R1—R2+3 not hyaline, ending at midway on Dc; Dc about two-fifths as long as Mc and 0.13× length of forewing. Base of fork 1, fork 2 and crossvein R3—R4 hyaline. Crossvein R—M hyaline, crossvein M1+2—M3+4 forming hyaline continuation of crossvein R—M; base of Mc forming almost triangular hyaline area. Cu1a almost straight, except weakly curving proximally and distally; distal part of Cu2 and arc hyaline. Posterior margin with row of anteriorly oriented setae in row about 1.5× longer than Dc. Genitalia (Figs 15–19): In lateral view, segment IX divided into high anterodorsal part and high posteroventral part separated from each other by narrow bridge (Fig. 15); anterodorsal part almost V-shaped in lateral view; posteroventral part of segment IX slightly shorter than anterodorsal part, as long and high as coxopodites in lateral view (Fig. 15). In lateral view, coxopodites originating as linear continuation of posteroventral plate of segment IX, parallelogram-shaped, with posterodorsal corner more strongly produced posteriorly; dorsal margin convex; posterior margin almost straight (Fig. 15); in ventral view, with each side diverging posteriorly, inner margin V-shaped (Fig. 17). Harpagones about half as long and nearly as high as coxopodites, divided into high dorsal branch with rounded apex, and short ventral branch with rounded apex; dorsal branch oriented posterodorsally, ventral branch oriented posteriorly; in ventral view rounded, club-shaped (Fig. 17). Tergum X oriented posteriorly, slender along its length in lateral view, apex narrow (Fig. 15); in dorsal view (Fig. 16) uniformly narrowing along its length; thick setae absent. Phallus (Figs 18, 19) longer than sum of segments IX and tergum X; with two small, weakly curving sclerites located at anterior and posterior third, respectively; anterior fourth globular, central two-fourth parallel-sided, half as high as anterior part, apical one-fourth low, parallel-sided in lateral view, narrowing in ventral view (Fig. 19).

#### Etymology.

*Gunungiella hueensis* was named after Hue City in which the type locality is situated.

### Gunungiella phulocensis sp. nov.

Taxon classificationAnimaliaTrichopteraPhilopotamidae

23CD563B-6476-591F-BEDA-B454D578D1A5

https://zoobank.org/A2E9F16B-FF26-4EE9-9628-54F5B312F0DB

Figs 4, 20–24

#### Type material.

**Holotype**. Vietnam • ♂; Thua Thien Hue Province, Phu Loc District, Bach Ma National Park, stream, 16.2292°N, 107.8483°E, 363 m asl, Malaise trap, 22–29.iii.2023, loc#VN23-01, leg. KA Johanson & T Vu, VNMN in alcohol.

#### Diagnosis.

The male genitalia of *Gunungiella phulocensis* sp. nov. exhibit a notable similarity to those of *G. achtatrimchi* Schmid, 1968 from the Assam region in India, particularly in the posteriorly produced ventral corner of the coxopodites and the presence of several long, thick setae on tergum X. *Gunungiella phulocensis* sp. nov. is separated from *G. achtatrimchi* in dorsal view of the genitalia by having the apex of tergum X with a shallowly incision, not deeply incised as in *G. achtatrimchi*; and in lateral view, the harpagones are oriented posteriorly in *G. phulocensis* sp. nov., not dorsally as in *G. achtatrimchi*.

#### Description.

**Figures 20–24.**
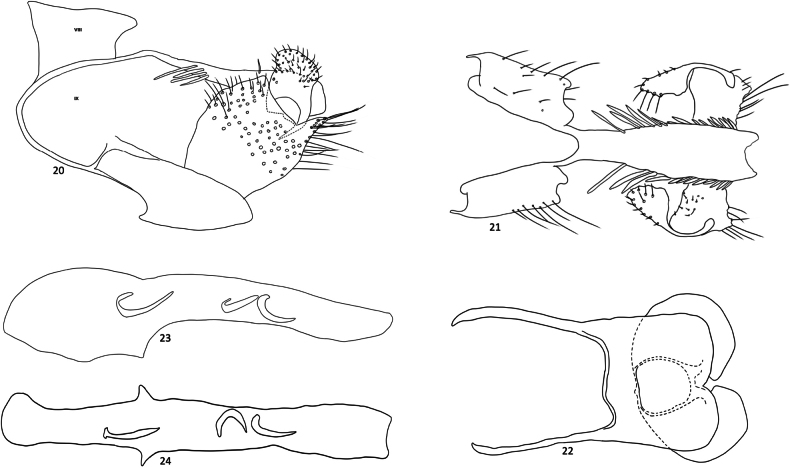
Male genitalia of the holotype of *Gunungiella phulocensis* sp. nov.; **20**. Lateral view; **21**. Dorsal view; **22**. Ventral view; **23**. Phallus, lateral view; **24**. Phallus, ventral view.

Forewing (Fig. 4) 3.6 mm (*N* = 1), membrane pale brownish, veins dark brown. Fork 1, 2 and 5 present; fork 1 originating distally at anterior corner of Dc; fork 2 originating slightly more basally than fork 1; crossvein R1—R2+3 not hyaline, ending at midway on Dc; Dc about two-fifths as long as Mc and 1.2× length of forewing. Base of fork 1, fork 2 and crossvein R3—R4 hyaline. Crossvein R—M hyaline, crossvein M1+2—M3+4 forming hyaline continuation of crossvein R—M; base of Mc forming almost triangular hyaline area. Cu1a slightly and uniformly curving; distal part of Cu2 and arc hyaline. Posterior margin with row of anteriorly oriented setae in row about 2× longer than Dc. In lateral view, abdominal segment VIII forming high, almost rectangular plate situated above thin anterior part of segment IX (Fig. 20). Genitalia (Figs 20–24): In lateral view, anterior part of segment IX forming slender, U-shaped, sclerotized strip between base of tergum X and high posteroventral part of segment IX (Fig. 20); posteroventral part of segment slightly longer than coxopodites in lateral and ventral view (Figs 20, 22), forming almost rectangular plate in ventral view (Fig. 22); in ventral view almost rectangular. Coxopodites originating subapically from segment IX, oriented posterodorsally, with convex dorsal and ventral margins in lateral view (Fig. 20); posterior margin straight except posteroventral corner strongly produced posteriorly (Fig. 20). Harpagones about half as long and one-third as high as coxopodites, almost rectangular, except narrowing at mid-length in lateral view; oriented dorsally along their length. Tergum X slender in lateral view, gradually thickening apically, apex truncate (Fig. 20); in dorsal view (Fig. 21) widening distally at anterior half, posterior half about parallel-sided; each side with thick setae in two rows: anterior row with about four anteroventrally oriented setae about as long as width of tergum X; posterior row with about ten setae circa half as long as anterior setae, hidden in lateral view behind coxopodites; oriented anteroventrally. Phallus (Figs 23, 24) longer than sum of segments IX and tergum X; with three about equally long, curving sclerites located in middle; anterior two-fifths about twice as high as posterior three-fifths; apex rounded in lateral view and truncate in ventral view (Fig. 24).

**Figure 25.**
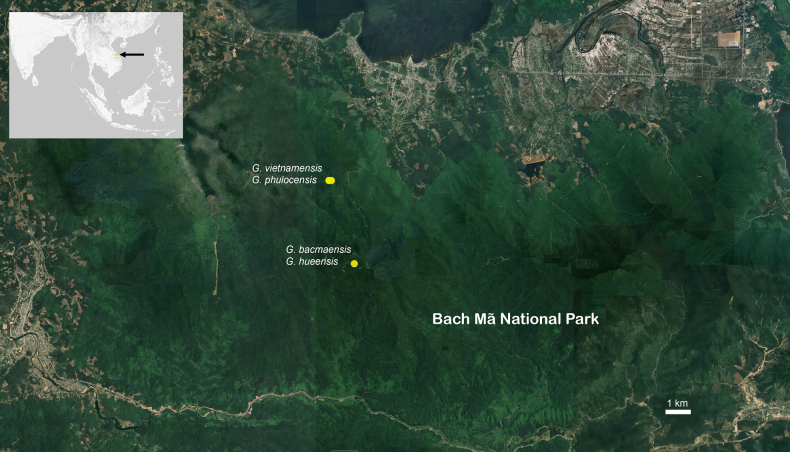
Map of Bạch Mã National Park showing the locations of the collection sites for the four new *Gunungiella* species. The pale inset map shows the very approximate area of distribution for the genus *Gunungiella* as well as the position of the national park (arrow).

#### Etymology.

*Gunungiella phulocensis* was named after the Phu Loc commune in Hue City where the type locality is situated.

### Gunungiella sosau

Taxon classificationAnimaliaTrichopteraPhilopotamidae

Oláh & Malicky, 2010

5424AE0D-3B71-57DC-B3AC-BA2B74131DBE

#### New record.

Vietnam • ♂; Cao Bang Province, Nui Pia Oac Nature Reserve, about 2.5 km S Na Reo Village, about 500 m S start of aqueduct, Malaise trap across Khuoi Dac Stream, bottom with stones and gravel, 8–10.iv.2012, loc#VN021, leg. Johanson & Pham [NHRS].

##### Key to males of known *Gunungiella* species in Vietnam

**Table:** 

1	In lateral view of genitalia, harpagones lowest at mid-length	**2**
–	In lateral view of genitalia, harpagones highest at mid-length	**5**
2	In lateral view of genitalia, coxopodites with posteroventral corner sharply produced posteriorly	**3**
–	In lateral view of genitalia, coxopodites with posteroventral corner rounded, not produced posteriorly	**4**
3	In lateral view of genitalia, coxopodites with posterodorsal corner produced posteriorly into long, curving plate; segment IX with pair of long, posteriorly oriented branches; tergum X without stout setae in rows	***G. dung* Oláh & Malicky, 2010**
–	In lateral view of genitalia, coxopodites with posterodorsal corner not produced posteriorly; segment IX without posteriorly oriented branches; tergum X with stout setae in rows	***G. phulocensis* sp. nov**.
4	In lateral view of genitalia, coxopodites with convex dorsal margin; harpagones club-shaped with apex pointed ventrally	***G. cao* Oláh & Malicky, 2010**
–	In lateral view of genitalia, coxopodites with undulating dorsal margin; harpagones each with posterodorsal corner strongly produced	***G. obendio* Oláh & Malicky, 2010**
5	In genitalia, segment IX with long, posteriorly oriented processes	**6**
–	In genitalia, segment IX lacking posteriorly oriented processes	**10**
6	In genitalia, segment IX with pair long, posteriorly oriented, unbranched processes	**7**
–	In genitalia, segment IX with long, bifurcated, posteriorly oriented processes	**9**
7	In genitalia, phallus almost straight in lateral view	***G. hueensis* sp. nov**.
–	In genitalia, phallus almost uniformly curving ventrally in lateral view	**8**
8	In genitalia, tergum at mid-length more than half as high as the coxopodites	***G. lencao* Oláh & Malicky, 2010**
–	In genitalia, tergum at mid-length less than one-third as high as the coxopodites	***G. vietnamensis* sp. nov**.
9	In genitalia, segment IX with dorsal processes with long, thick dark at apex; tergum X reaches as far posterior as apex of harpagones	***G. bacmaensis* sp. nov**.
–	In genitalia, segment IX with dorsal processes without setae; tergum X reaches only end of coxopodites	***G. bongai* Oláh & Malicky, 2010**
10	In genitalia, tergum X almost reaches posterior apex of coxopodites	***G. sosau* Oláh & Malicky, 2009**
–	In genitalia, tergum X reaches about mid-length on coxopodite	**11**
11	In genitalia, harpagones pointed posteriorly	***G. arinada* Oláh & Malicky, 2010**
–	In genitalia, harpagones rounded posteriorly	**12**
12	In genitalia, tergum X widening before apex; phallus with four internal spines	***G. thuba* Oláh & Malicky, 2010**
–	In genitalia, tergum X parallel-sided along its length; phallus with five internal spines	***G. guni* Malicky, 2009**

## Discussion

Representatives of the genus *Gunungiella* are recorded from India and Sri Lanka in the west, to Timor in south-east, and the distribution area includes China, Thailand, Vietnam, other parts of Indonesia, the Philippines and Malaysia (Morse 2026). Despite the relatively wide distribution and many species described (about 90 species with this report), the discovery of four new *Gunungiella* species within a geographically restricted area suggests that the diversity of the genus may be underestimated across other parts of its known distribution area. This interpretation is partly based on the previous records from Vietnam (Oláh and Malicky 2010), where two species (*G. bongai* and *G. lencao*) were collected from the Duchma Stream and two species (*G. cao* and *G. dung*) from the Baco Stream. Support for the idea of underestimation also came from Schmid (1968), who hypothesized that the genus holds some 400 species worldwide. Several previously described Vietnamese species, including *G. arinada*, *G. bongai*, *G. dung*, *G. lencao*, and *G. obendio*, have been recorded from tributaries of larger streams and brooks (Oláh and Malicky 2010). Similarly, all four newly described species were collected from well-oxygenated streams with moderate to high flow velocities, suggesting that *Gunungiella* species may be associated with fast-flowing lotic habitats, including smaller water courses. It seems that the Vietnamese *Gunungiella* species are associated with the upper parts of running water. Despite a predominance of diversity in the upper parts of streams, Schmid (1968) reported species also from medium-sized to large, fast-flowing rivers and even the foot of waterfalls.

## Supplementary Material

XML Treatment for Gunungiella

XML Treatment for Gunungiella vietnamensis

XML Treatment for Gunungiella bacmaensis

XML Treatment for Gunungiella hueensis

XML Treatment for Gunungiella phulocensis

XML Treatment for Gunungiella sosau

## Additional information

### Artificial Intelligence (AI) use

The authors accept full responsibility for the content of the manuscript, including the disclosure of any use of AI.

No AI tools were used in the preparation of this manuscript.

### Author ORCIDs

K.A. Johanson https://orcid.org/0000-0002-1893-3429

H.T. Pham https://orcid.org/0000-0002-4763-3679

### Data availability

All of the data that support the findings of this study are available in the main text.
